# Inverse design of optical lenses enabled by generative flow-based invertible neural networks

**DOI:** 10.1038/s41598-023-43698-3

**Published:** 2023-09-29

**Authors:** Menglong Luo, Sang-Shin Lee

**Affiliations:** Department of Electronic Engineering, Kwangwoon University, Seoul, 01897 Republic of Korea

**Keywords:** Optical techniques, Computational science, Computer science

## Abstract

Developing an optical geometric lens system in a conventional way involves substantial effort from designers to devise and assess the lens specifications. An expeditious and effortless acquisition of lens parameters satisfying the desired lens performance requirements can ease the workload by avoiding complex lens design process. In this study, we adopted the Glow, a generative flow model, which utilizes latent Gaussian variables to effectively tackle the issues of one-to-many mapping and information loss caused by dimensional disparities between high-dimensional lens structure parameters and low-dimensional performance metrics. We developed two lenses to tailor the vertical field of view and magnify the horizontal coverage range using two Glow-based invertible neural networks (INNs). By directly inputting the specified lens performance metrics into the proposed INNs, optimal inverse-designed lens specifications can be obtained efficiently with superb precision. The implementation of Glow-assisted INN approach is anticipated to significantly streamline the optical lens design workflows.

## Introduction

Over the preceding decades, optical lenses have become increasingly valuable from a commercial standpoint and are extensively utilized in diverse applications, including beam shaping^1^, antennas^2,3^, autonomous vehicles^4,5^, and virtual reality products^6,7^. Obtaining an optical lens system of superior quality necessitates a complex design process and extensive refinement, thus requiring substantial dedication of time and effort. Customary procedures for the design of optical lenses entail the creation of lens surface data along with the determination of dimension and spatial location details. This process evaluates several crucial factors, such as the surface radius of curvature, thickness, working distance, conic constant, and manufacturing material. In practical engineering problems, the desired lens performance metrics is typically preestablished, whereas the structural parameters of the lens are unknown. When confronted with specific requirements, such as prescriptive dimensions for beam width and deflection angle area, engineers are supposed to obtain suitable lens parameters that satisfy the specified objectives. Although currently available commercial optical software offers certain optimization functions, the duration of the optimization process may vary from tens of minutes to several hours. Additionally, optimization software may converge to local optima, resulting in failure to produce the optimal outcomes and thus requiring further optimization iterations. This negatively affects processing time and complexity. Automatically inferring lens specifications from given performance requirements can substantially diminish the intricacy and development time of the lens design process. Therefore, our research aimed at conceiving an efficient solution to autonomously generate lenses that satisfy the required performance through the application of deep learning techniques, thereby streamlining and expediting lens design.

However, the conventional fully connected neural networks exhibit deficiency in predictive precision when applied to the inverse lens design. In our previous study^8^, a multilayer perceptron (MLP) architecture was attempted to serve as the framework of a fully connected neural network for the inverse lens design. The lens performance metrics were used as input features while the structural parameters were adopted as output labels for the MLP. In practice, the accuracy of the predictions generated by the MLP was found to be as low as approximately 30%. Performing the lens design utilizing the MLP required a trial-and-error process, in conjunction with subsequent analysis facilitated via a database server MySQL to identify the optimal lens structures. Furthermore, additional applications^9^ of machine learning methods for the lens system development predominantly provide initial approximate configurations rather than optimal lens specifications. Inverse design problems are deemed to be ill-posed^10–12^, signifying that they are commonly acknowledged to exhibit the characteristic of one-to-many mapping. That is, different combinations of lens structural parameters may yield the same lens performance metrics. The inherent one-to-one mapping nature of conventional regression algorithms poses a challenge in dealing with the complexity of one-to-many mapping. Moreover, the MLP cannot mitigate information loss resulting from dimensional difference between the lens performance metrics and structural parameters, thus lacking the capability of deducing specific lens structures exclusively from the performance metrics. In this context, a more competitive deep learning approach leading to precise specifications for the inverse-designed lenses is highly desirable. Invertible neural networks (INNs), which are based on the flow models, were first introduced by Ardizzone et al.^10^. The flow models have undergone a series of progressive evolutions and refinements stemming from nonlinear independent components estimation^13^ and advancing toward real-valued non-volume preserving transformations^14–17^. This progression culminated with the development of an evolved generative flow model, known as Glow^18–21^. As technology breakthroughs have endowed invertible models with enhanced capabilities to effectively tackle intricate inverse problems, INNs are purposefully engineered to provide a precise inverse mapping for each of the forward mapping. Therefore, the INNs not only can infer the outputs from the inputs but they can also reconstruct the initial inputs from the outputs without loss of information. The applications of INNs have triggered an upsurge in the exploration of inverse design across several scientific investigations, including innovative materials^22^, aerosols development^23^, multiphase flow^24^, and imaging^25–27^. Nevertheless, inverse design applications in the field of optical lenses remain limited. Incorporating Glow-based INNs into lens development can facilitate effective inverse design of lens systems by directly predicting the numerous parameters of lenses, consequently streamlining the research and exploration of these devices. As a result, our intention aligns with the utilization of Glow-based INNs with the intention of implementing the inverse design methodology for optical lenses.

In this study, we addressed the inverse design of lenses efficiently by leveraging two Glow-based INNs. It has been confirmed that our models yield a sufficiently high level of precision. Furthermore, the models enable the prompt derivation of lens specifications that closely match the intended functionalities of the proposed converging and beam-coverage enhancing lenses. The combination of Glow-based INNs and inverse lens design is a prominent scheme for simplifying the lens design workflows. The proposed models can be regarded as potent inverse modelling tools providing creators with reliable lens structural parameters to solve the practical requirements of lens design.

## Structures of the proposed converging and beam-coverage enhancing lenses

**Figure 1:**
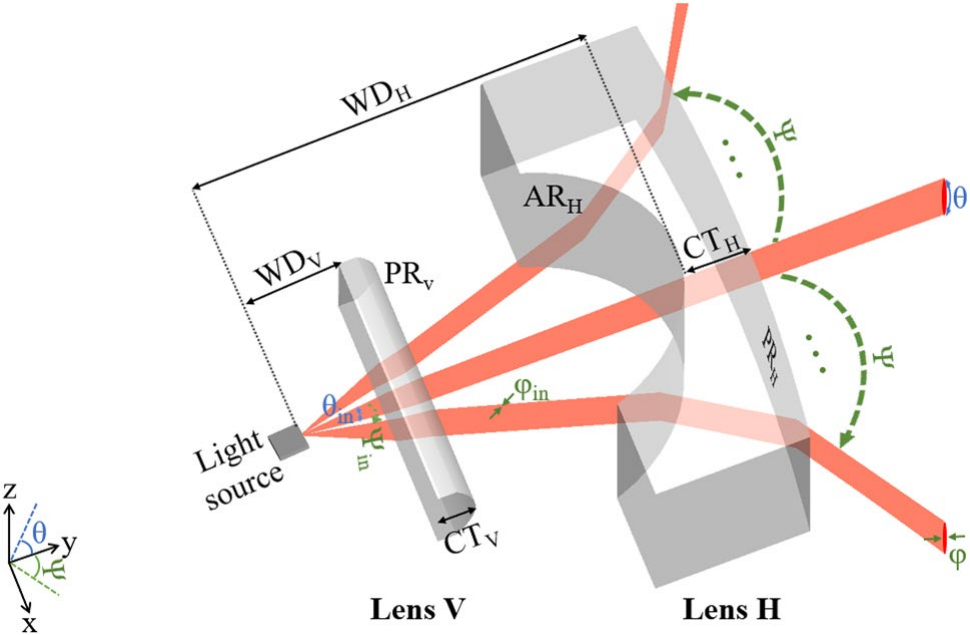
Configuration of the proposed lens module and ray tracing associated with the lens functionalities. Lenses V and H are devised to diminish vertical beam divergence and amplify horizontal beam coverage, respectively.

In this work, we attempted to embody an optical module comprising converging and beam-coverage boosting lenses, denoted as lenses V and H, respectively. Our goal was to develop lens V to curtail the beam divergence observed along the vertical orientation, and lens H to concurrently augment the horizontal propagation range of the beams emitted from a light source. Notably, the proposed Glow-based INN technique holds the significant potential for the inverse design of various types of lenses. In consideration of multifarious nature of actual engineering situations encountered in lens design concern, such as the use of light sources possessing diverse beam divergence angles and different lens manufacturing materials. Therefore, our study focused on the implementation of lenses made of diverse materials capable of accommodating varying beam divergence angles. Herein, two full-width-at-half-maximum angles, namely 20° and 25°, were adopted as the vertical beam divergence θ_in_ of the light source, respectively. In addition, two categories of materials were selected for the lens system, specifically polycarbonate (PC) plastic and HZF6 glass. The refractive indices of PC and HZF6 at a working wavelength of 1550 nm are 1.56 and 1.72, respectively. The optical system has been subjected to meticulous scrutiny through a ray-optic tool, LightTools (LT, Synopsys Inc., USA). The illustration delineated in Fig. 1 portrays the trajectory of the beams as they sequentially traverse lenses V and H. The scope of our design is solely responsible for the operation of geometric optics rather than diffractive optics. The three beams illustrated in Fig. 1 are representative of their emission at distinct steering angles. The central beam corresponds to an input deflection angle of 0°, while the beam located at either end is indicative of the central beam which is being successively scanned over the horizontal coverage range. The diminution in the vertical field of view of the beams along the z-axis is concomitant with the expansion of the horizontal coverage range as the beams advance along the y-axis. The incident beams, characterized by the θ_in_, horizontal beam divergence φ_in_ of 0.23°, and maximum input horizontal coverage range Ψ_in_ of 15°, are emanated from the light source. These beams then transmit through the anterior surface of the lens V and subsequently exit through its posterior surface at a diminished angle of θ. The degree of reduction in the vertical field of view is contingent upon the structural parameters of the lens V, including the working distance WD_V_, center thickness CT_V_, conic constant CC_V_, and posterior radius of curvature PR_V_. After the lens V decreases the vertical beam divergence, the beams proceed through the lens H. The front concave surface of lens H increases the deflection angle of the beams, while the posterior surface reduces the steering angle slightly. To mitigate the potential for excessive growth in the horizontal output beam divergence φ, a convex configuration was chosen for the posterior surface of lens H, thereby alleviating the horizontal aberration. At the end of the process, the beams pass through the lens H with an output scanning range of Ψ. The horizontal deflecting magnification is reliant upon the parameters of the lens H, including the working distance WD_H_, center thickness CT_H_, and radii of both the anterior and posterior surfaces AR_H_ and PR_H_.

## Process of the inverse lens design relying on the proposed INNs

**Figure 2:**
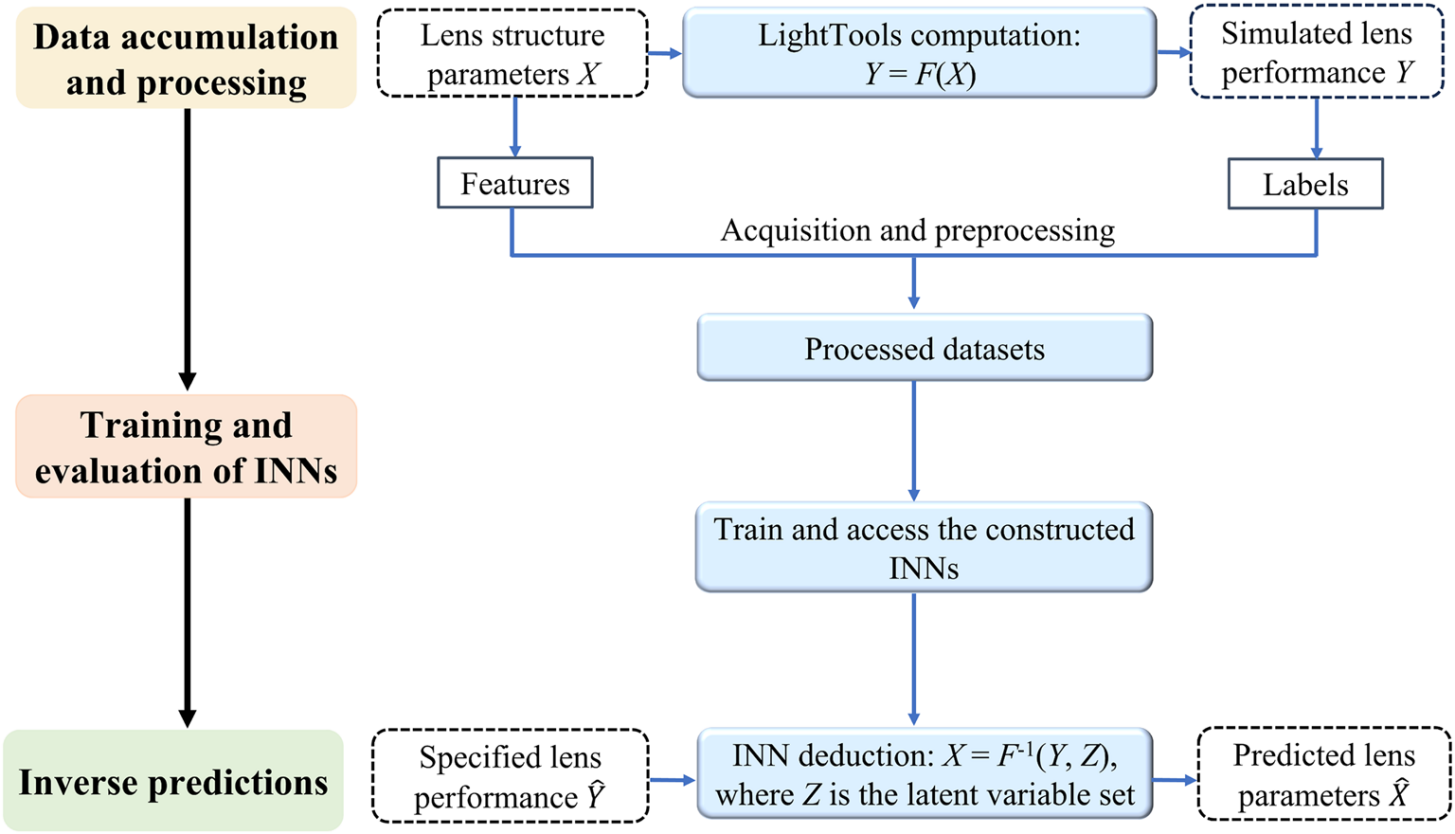
INNs-based inverse lens design workflow. The process includes three steps: accumulation and processing of data, training and evaluation of the proposed INNs, and inverse predictions of lens parameters.

The process of the proposed INN-based inverse design of lenses encompasses a sequence of operations. The initial step is data acquisition and pre-processing, which plays a crucial role in ensuring the effectiveness and accomplishment of deep learning scheme. In this work, the datasets for lenses V and H were obtained through LT simulations. The data collection was fulfilled by taking into account the predetermined lens parameters as the set of features *X* for each dataset, while the performance parameters were assigned as the relevant labels *Y*. Subsequently, the pre-processed data were fed into the INNs for both model training and testing purposes. Following the completion of the training process, the required lens performance metrics were inputted into the INNs, which then inferred the corresponding optimal lens parameters. The entire workflow is described in Fig. 2. The implemented INNs were coded in Python and executed on a computer system featuring an Intel Core i5-10400 processor, an NVIDIA GeForce GTX 1650 SUPER graphics card, and a memory capacity of 32 GB.

### Data acquisition and preliminary processing

The objective of constructing the INNs for the lens system is to derive the structural parameters of the lenses directly and autonomously by specifying a set of lens performance metrics encompassing θ, Ψ, and φ. Considering that certain elements, such as the dimensions of the lens in terms of height and width, have no substantial influence on the optical performance of the lens system, the input features *X* of the proposed INNs were determined to be the working distance, center thickness, conic constant, and radius of curvature. The labels *Y* collected for the INNs correspond to the lens performance metrics, including θ of the lens V, as well as Ψ and φ values of the lens H. To derive the features and labels, the parameter sweep tool integrated in LT was exploited. The ranges of the acquired feature values were established a priori based on previous expertise in lens design. After defining the features, the data collection instrument obtained the corresponding labels by computing the diversified combinations of features. Tables 1 and 2 provide an overview of the features that pertain to the datasets of lenses V and H, respectively. Before feeding the raw datasets into the models, the datasets were pre-processed. To ensure the dependability of the proposed INNs, a randomized shuffle operation was executed on the datasets, followed by the segregation of the datasets into two subgroups, one for training and one for testing, with an allocation ratio of 80% and 20%, respectively. Subsequently, we adopted StandardScaler, a class offered by the scikit-learn library, to standardize the features in the dataset (i.e., transforming the features in the dataset into a standardized normal distribution). This conversion serves to promote data comparability across various features, thereby streamlining both the learning and optimization processes, while simultaneously augmenting the accuracy and effectiveness of the models. In adherence to the method of standardization, the process of manipulating each feature of the training set entails the computation of $z = \frac{{x {-} \mu }}{s}$, where *x* denotes the training samples associated with each feature, while *μ* and *s* are the mean and standard deviation of the training samples, respectively. The process of centering and scaling involves individual computation of relevant statistics on the samples within the training set for each feature. After standardization, the distribution of each feature leads to a mean value of zero and variance value of one. The resultant mean and standard deviation values were saved and used for subsequent processing of the test dataset.

**Table 1:** Ranges of the features pertaining to the datasets of the lens V.

Features	Minimum	Maximum	Increment
WD_V_ (mm)	1	7	0.2
CT_V_ (mm)	3	5	0.5
CC	–1	0	0.5
PR_V_ (mm)	4	10	0.1

**Table 2:** Ranges of the features of the datasets of the lens H.

Features	Minimum	Maximum	Increment
WD_H_ (mm)	24	40	1
CT_H_ (mm)	3	5	1
AR_H_ (mm)	5	10	0.125
PR_H_ (mm)	50	100	10

### Architecture of the proposed INNs for inverse lens design

**Figure 3:**
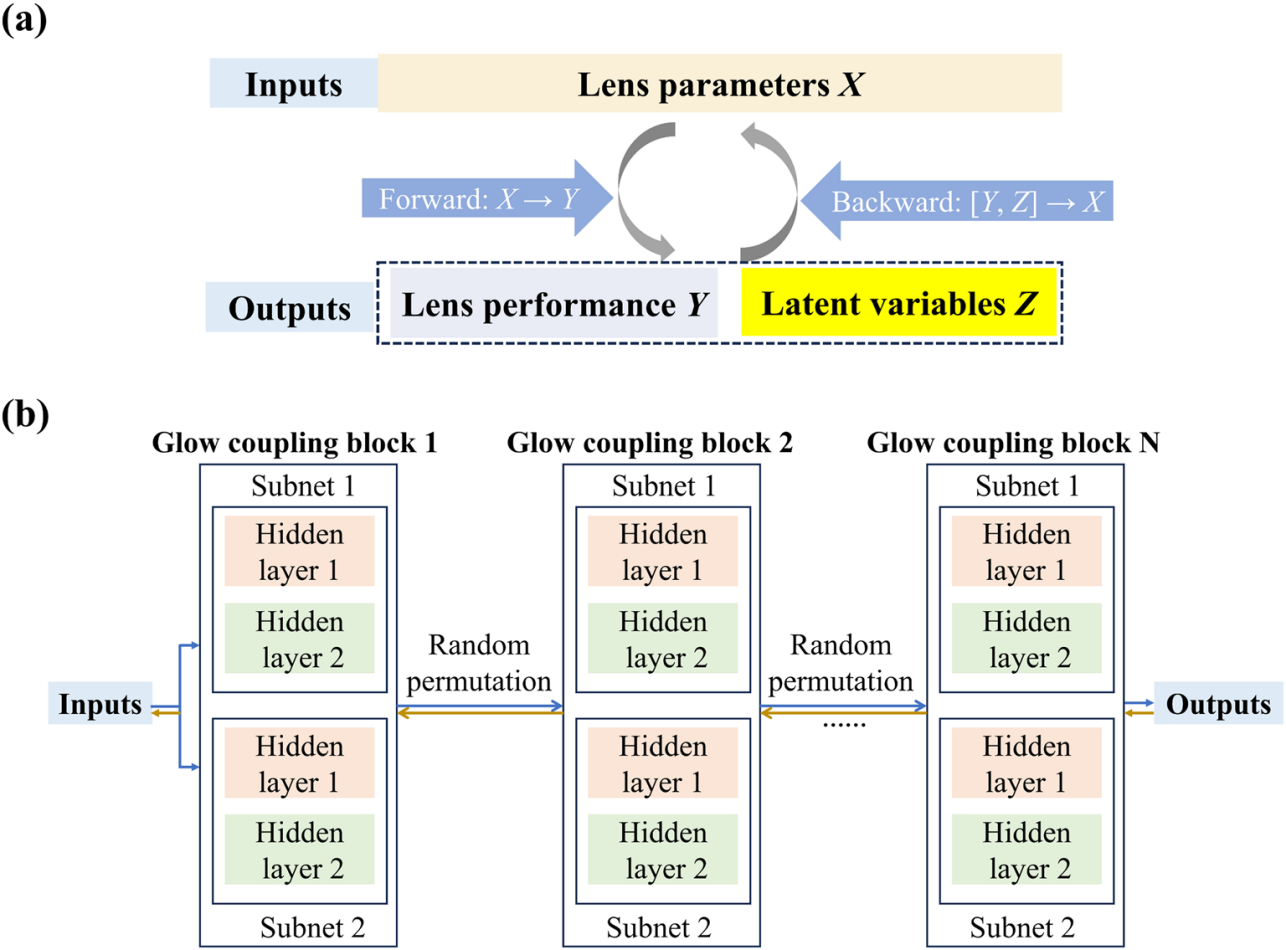
(**a**) Forward and backward mappings between the lens structural parameters *X*, performance *Y*, and latent variables *Z*. (**b**) Structure of the proposed INNs based on the Glow coupling blocks. The numbers of block N in the models for lenses V and H are eight and ten, respectively.

Considering that the intrinsic dimension of the labels *Y* is smaller than that of the input features *X*, the forward transformation process from *X* to *Y* may suffer from innate information loss^10^. To mitigate this, a latent variable set *Z* that conforms to a standard Gaussian distribution was introduced. Note that the dimension of set *Z* is equivalent to the dimensional difference between the sets *X* and *Y*. The use of variable set *Z* in the INNs ensures that information regarding *X* that is not encompassed within *Y* is captured effectively. Consequently, the correlation between the inputs *X* and outcomes *Y* is remodelled into a relationship between *X* and [*Y*, *Z*]. The forward operation represented by $F\left( X \right) = \left[ {Y, Z} \right]$ is accompanied by its corresponding inverse operation $X = F^{ - 1} \left( {Y, Z} \right)$. The forward and inverse mappings are expounded in Fig. 3a. The key aspect of an INN is to provide an architecture that ensures invertibility. Commonly applied strategies to construct the frameworks of INNs exploit affine coupling layers^10,12^, which divide the inputs *X* into two segments and apply invertible functions to a single segment only while keeping the other part unaltered. The affine transformation process engages the concatenation of multiplicative and additive coupling blocks, thereby leading to multifaceted transformation effects. The two INNs proposed for lenses V and H are composed of multiple Glow coupling block units interspersed with stochastic permutation operations, as depicted in Fig. 3b. The INNs facilitate the alternating execution of forward and reverse iterations during the training process, enabling bidirectional gradient propagation and allowing for subsequent parameter updates. To augment the nonlinear characteristics of the INNs, the PermuteRandom function was capitalized on to shuffle the data order randomly. Each Glow coupling block comprises two subnetworks, namely Subnets 1 and 2, which represent the bottom and top channels, respectively. The subnetworks are inclusive of sequential neural networks, each featuring two hidden layers with 256 fully connected neurons and the rectified linear unit (ReLU) activation function. The bottom channel initiates input data transfer to the latent space, whereas the top channel is responsible for the transfer of the latent space vector to the data space. Subnets 1 and 2 are exclusively allocated for computing outputs propagating through the lower and upper channel, respectively. The input lens parameters *X* undergo a division into two equal segments of length 2 for each coupling block. During the coupling transformation, a portion of the input data is sent to Subnet 1 for transformation, whereas the remaining portion remains unchanged. Subsequently, the unmodified portion is merged with the transformed data to give the outputs. The input data undergo both forward and backward propagation during training. Owing to the reversibility of the network, the outputs can experience backward propagation. Consequently, the results obtained by backward processing can be close to the inputs.

The INNs enable the sequential execution of forward and backward iterations during training, thus permitting the propagation of gradients in both directions and the subsequent updating of hyperparameters. Each coupling block featured a clamp value of 2, which could be leveraged to constrain the range of outputs for the bottom channel. In addition, the Adam optimizer was chosen for the reversible training with a learning rate of 1E–3 and weight decay rate of 1E–5. To ensure stable and reversible training, minor perturbations characterized by the standard deviation σ of Gaussian noise were introduced. Specifically, a standard deviation σ_y_ of 5E–3 was allocated to *Y* and a σ_z_ of 2E–3 was assigned to *Z*. Furthermore, establishing loss functions is integral to guarantee the effective training of the models. The total loss function is expressed as $L_{total} = \lambda_{y} L_{y} + \lambda_{z} L_{z} + \lambda_{x} L_{x}$, where $\lambda_{y}$, $\lambda_{z}$, and $\lambda_{x}$ were defined as the weighting factors that were all empirically determined to be one. *L*_y_ is the mean squared error (MSE) loss function, which imposes a constraint on the predicted outputs of the network during the forward propagation phase, thus ensuring that predictions remain within the range of ground truth data. Additionally, $L_{z}$ and *L*_x_ were realized using the maximum mean discrepancy (MMD) to quantify the disparity between the anticipated outputs and input values, thereby guaranteeing that the overall distribution of the outputs conforms to that of the actual values.

### Loss assessment of the proposed INNs on the datasets for lenses V and H

**Figure 4:**
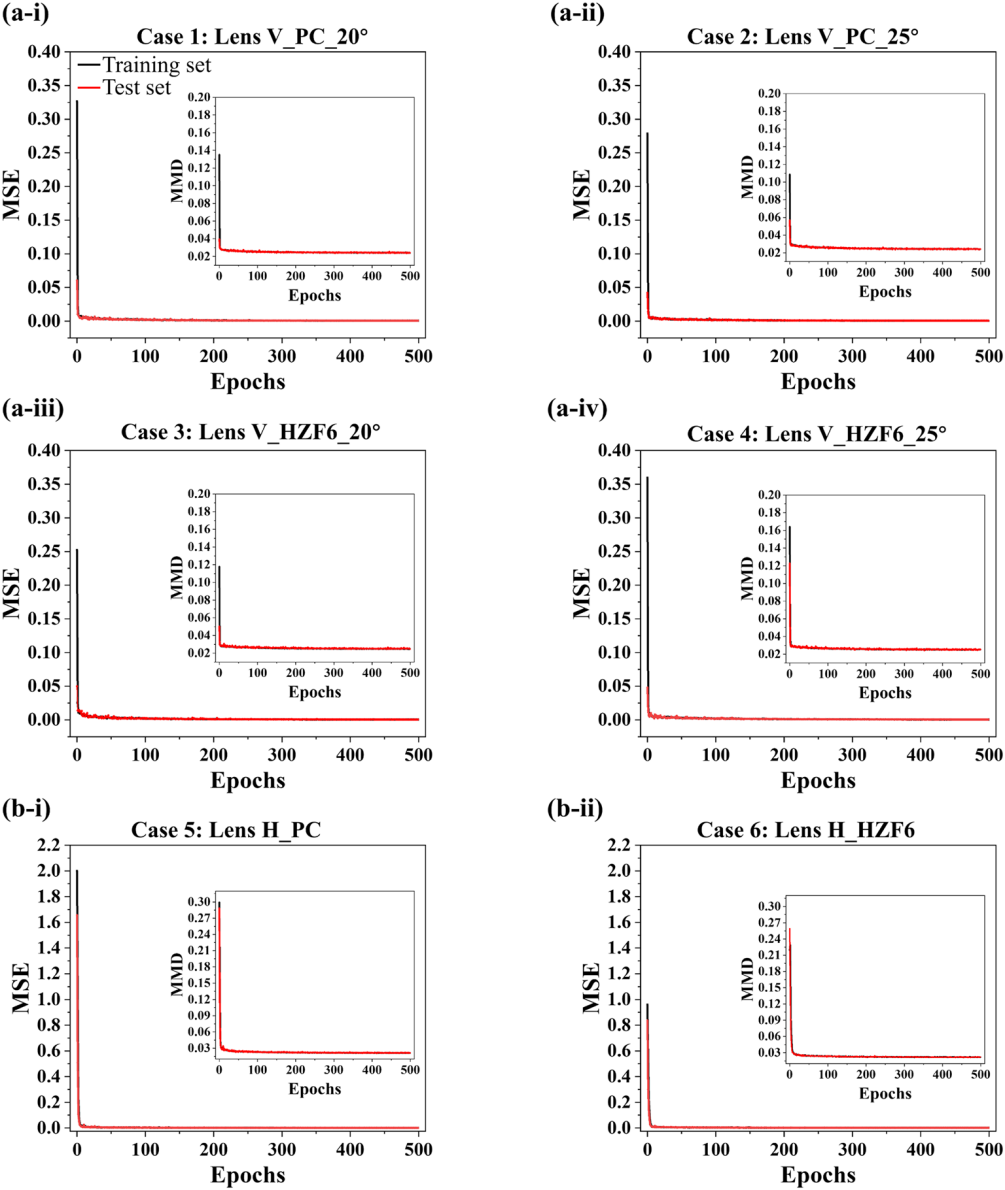
MSE pertaining to the lens performance metrics and MMD related to the lens structural parameters computed for six datasets over 500 epochs on both the training and test sets. The MSE and MMD values of the lens V were obtained considering (**a-i**) PC and θ_in_ = 20°, (**a-ii**) PC and θ_in_ = 25°, (**a-iii**) HZF6 and θ_in_ = 20°, and (**a-iv**) HZF6 and θ_in_ = 25°. The MSE and MMD of lens H were obtained using (**b-i**) PC and (**b-ii**) HZF6.

In this work, the effectiveness of the proposed two INNs for lenses V and H was checked by taking advantage of four and two datasets, respectively. The construction of the four datasets pertaining to the lens V involved PC and HZF6 materials, under θ_in_ values of 20° and 25°. Similarly, the two datasets associated with the lens H were created based on the same materials. The four cases for the lens V are labelled as Lens V_PC_20°, Lens V_PC_25°, Lens V_HZF6_20°, and Lens V_HZF6_25°, while the two cases for the lens H are named as Lens H_PC and Lens H_HZF6. The runtime overheads for the six cases of model training are 2038, 1825, 1820, 1416, 1511, and 820 s, respectively. The disparity in runtime may be ascribed to the varying size of the datasets, which results from the exclusion of parameter combinations of lens structures that would yield bimodal non-Gaussian beam profiles in the vertical direction and excessively broad coverage range in the horizontal direction. It is noted larger datasets tend to incur longer runtime for the model training. Throughout the training phase, the MSE of lens performance metrics and the MMD of lens structural parameters on both the training and testing sets were plotted for each epoch. MSE is a widely adopted metric in statistics and machine learning fields that quantifies the difference between predicted values and ground-truth data. It serves as a reliable tool for evaluating the prediction accuracy of a model. Specifically, the lower the MSE, the higher is the accuracy of the predictions of the model. The MSE is defined as follows: $MSE \left( {Y, \hat{Y}} \right) = \left( \frac{1}{n} \right) \times \sum \left( {y_{i} {-} \hat{y}_{i} } \right)^{2}$, where *n* represents the sample size, $y_{i}$ denotes the performance values computed in LT, and $\hat{y}_{i}$ represents the performance requirements conveyed into the INNs. Additionally, MMD is popularly utilized to quantify the dissimilarities between the outputs produced by a network and those obtained from empirical observations, thereby gauging the similarity between the two kinds of data. The diminution of MMD signifies a heightened resemblance between the expected and factual values. MMD is calculated as follows: $MMD\left( {X, \hat{X}} \right) = \left[ {\frac{1}{{n^{2} }}\mathop \sum \limits_{i}^{n} \mathop \sum \limits_{{i^{\prime}}}^{n} k\left( {x_{i} ,x_{i}{\prime} } \right) - \frac{2}{nm}\mathop \sum \limits_{i}^{n} \mathop \sum \limits_{j}^{m} k\left( {x_{i} ,\hat{x}_{j} } \right) + \frac{1}{{m^{2} }}\mathop \sum \limits_{j}^{m} \mathop \sum \limits_{{j^{\prime}}}^{m}k\left( {\hat{x}_{j} ,\hat{x}_{j^{\prime}} } \right)} \right]^{\frac{1}{2}}$, where $X$ and $\hat{X}$ correspond to the actual and predicted sets, respectively, and *k* corresponds to the Gaussian kernel function. The MSE values for each θ computed for the lens V on four datasets over 500 epochs are shown in Fig. 4a-i–a-iv, while those of Ψ and φ computed for the lens H on two datasets are plotted in Fig. 4b-i and b-ii, respectively. Additionally, the MMD values for lens parameters for both the training and test sets within 500 epochs are plotted in the subgraphs of Fig. 4. In the cases of lens V, the initial MSE values for θ on both the training and testing sets began at approximately 0.36 and 0.06, respectively, as indicated in Fig. 4a. Similarly, the highest MMD values for the lens parameters on both the training and test sets initiated at around 0.15 and 0.1, respectively, as depicted in the subfigures of Fig. 4a. The MSE and MMD decreased with an increasing number of epochs and ultimately stabilized at the vicinity of 0.001 and 0.025, respectively. During the early phases of training, the MSE observed in the training set surpassed that of the testing set. This can be attributed to the inability of the networks to comprehensively ascertain the underlying features of the data at the outset. Through training and optimization, the models acquired an understanding of the data characteristics, leading to a gradual decrease in the MSE of both the training and test sets. When the MSE of the training and test sets stabilized, the models effectively fitted the trained data, thereby allowing for the generalization to fresh data. An analogous trend can be discernible for the MSE and MMD of the lens H in Fig. 4b-i–b-ii. Both the MSE and MMD values declined from the values of 2 and 0.3 as the number of epochs increased, and ultimately plateaued at approximately 0.001 and 0.022, respectively. Both INNs assigned to the lenses V and H gave rise to a considerable level of precision, as evidenced by the significantly low MSE and MMD values achieved across the six datasets. Also, the proposed INNs have provided substantial versatility in accommodating diverse materials and light sources with distinct input beam divergences, thereby underpinning the extensive applicability of our INN-based approach.

### Reliability verification of the lenses V and H deduced by the proposed INNs

**Figure 5:**
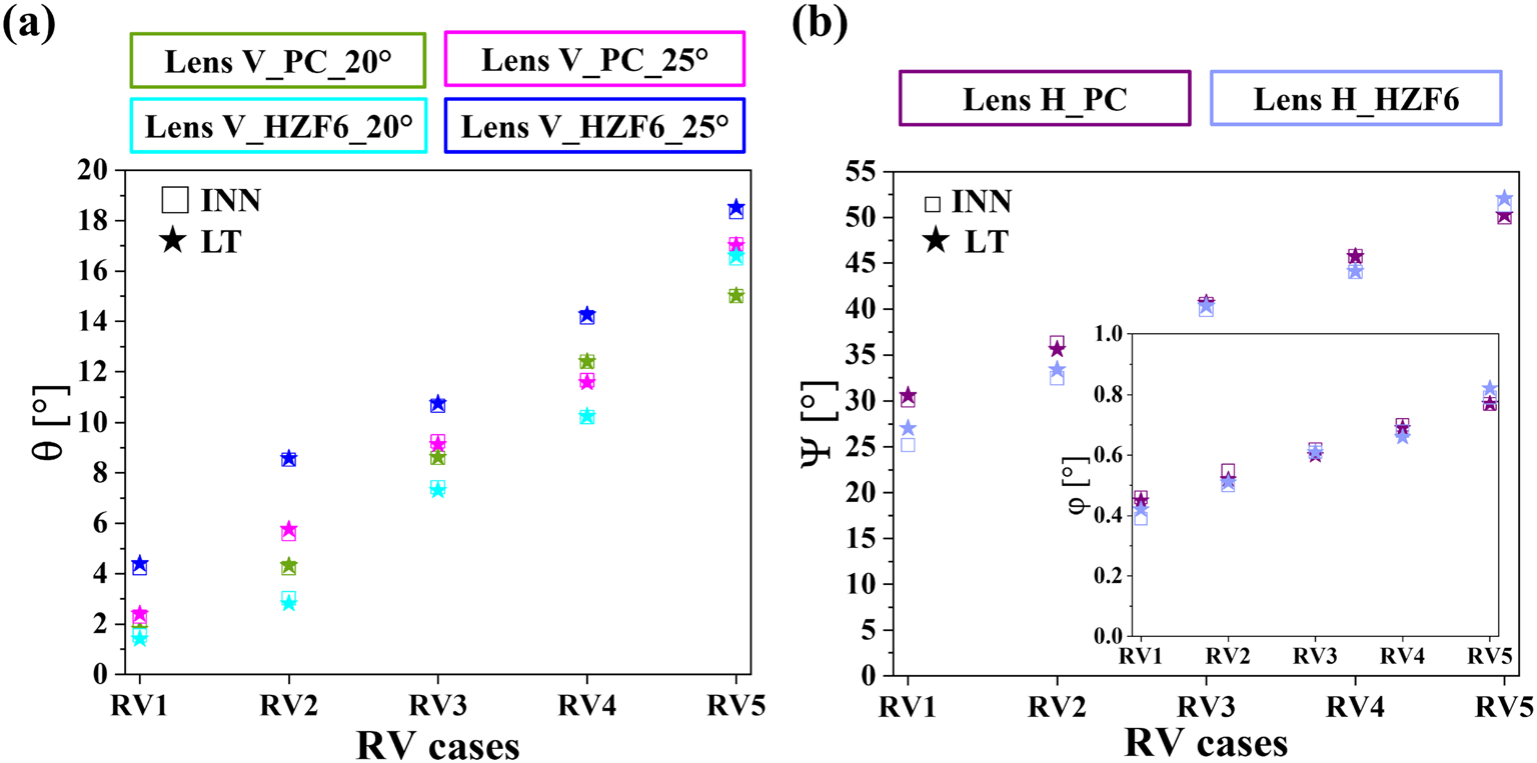
Comparison between the lens performance metrics inputted into the INNs and the corresponding LT outcomes for lenses V and H under five different RV scenarios. (**a**) Disparities between the assigned θ and the values generated by LT for lens V. (**b**) Difference between the designated Ψ and φ and the simulation results obtained using LT for lens H.

The reliability of the proposed INNs has been particularly substantiated in terms of the MSE and MMD values. After training, the two INNs were applied to accurately forecast the specifications of lenses V and H satisfying the designated lens performance metrics. To validate the proposed INNs under practical scenarios, a series of randomly selected performance metrics, encompassing θ for lens V as well as Ψ and φ for lens H, were inputted into the INNs. The primary objective of fulfilling the random verifications (RVs) is to concretely underpin the precision of the trained INNs. The proposed models were tested by conducting RVs accordingly on the six datasets of lenses V and H. θ of lens V was randomly assigned to five values. Similarly, five sets of stochastic values were allocated to Ψ and φ of lens H. A comparison was conducted between the performance metrics specified in the INNs and those obtained from LT simulations based on the predicted lenses, as expounded in Tables 3 and 4. The minor divergence between the specified lens performance metrics and those obtained through LT simulations can indicate that the INNs possess dependability in predicting lens parameters. The values of θ, Ψ, and φ under the label of INN are the lens performance metrics specified for the proposed INNs. Once these performance values are fed into the trained INNs, the models can predict the corresponding lens structural parameters. The lens structural parameters deduced from the INNs are subsequently subjected to the testing in LT to assess their validity. The lens structural parameters are reflected in the LT simulations to yield corresponding outcomes for θ, Ψ, and φ, which are categorized under the tag of LT. Table 3 tabulates a comparative analysis of θ values inputted in the INN and those generated through LT simulations. The results imply that the differences between the two θ values fluctuate around 0.1°, with a maximum deviation of approximately 0.2°, which fall within the bias range and can be disregarded. Additionally, the comparison presented in Table 4 reveals that the values of Ψ and φ specified in the INNs and those obtained using LT match significantly, with deviations not exceeding 1° and 0.03°, respectively. The overall contrast of the performance metrics inputted to the INNs and outcomes calculated by LT simulations is depicted Fig. 5a and b, which visually delineate the exceedingly minute gap between the two sets of results. The lens structural parameters deduced from the two proposed INNs are showcased in Tables 5 and 6. Considering the lens parameters derived from the INNs are practically affordable from the perspective of manufacturing, the proposed INNs are confirmed to be efficacious in determining the lens structural parameters by tapping into the corresponding performance metrics. The proposed models effectively predicted the parameters of lenses V and H with a computational time below 0.2 s.

**Table 3:** Comparison of values of θ inputted into the INNs and computed by LT under five RVs.

Cases	Lens V_PC_20°		Lens V_PC_25°		Lens V_HZF6_20°		Lens V_HZF6_25°	
	θ							
	INN	LT	INN	LT	INN	LT	INN	LT
RV1	1.83°	1.80°	2.30°	2.40°	1.55°	1.41°	4.22°	4.40°
RV2	4.21°	4.32°	5.57°	5.76°	3.03°	2.82°	8.53°	8.58°
RV3	8.60°	8.62°	9.25°	9.13°	7.43°	7.31°	10.66°	10.76°
RV4	12.40°	12.40°	11.66°	11.57°	10.22°	10.25°	14.16°	14.26°
RV5	15.00°	15.01°	17.05°	17.01°	16.50°	16.59°	18.33°	18.52°

**Table 4:** Comparison of Ψ and φ inputted into the INNs and calculated by LT under five RVs.

Cases	Lens H_PC				Lens H_HZF6			
	Ψ		φ		Ψ		φ	
	INN	LT	INN	LT	INN	LT	INN	LT
RV1	30.11°	30.59°	0.46°	0.45°	25.20°	24.13°	0.39°	0.37°
RV2	36.33°	35.60°	0.55°	0.52°	32.46°	33.39°	0.50°	0.51°
RV3	40.52°	40.65°	0.62°	0.60°	39.89°	40.34°	0.61°	0.61°
RV4	45.79°	45.74°	0.70°	0.69°	44.06°	44.12°	0.68°	0.66°
RV5	50.00°	50.28°	0.77°	0.77°	51.35°	52.08°	0.79°	0.82°

**Table 5:** Predicted lens structural parameters [WD_V_ (mm), CT_V_ (mm), CC, and PR_V_ (mm)] corresponding to the θ values inputted into the INNs of lens V under different RVs.

Cases	Lens V_PC_20°	Lens V_PC_25°	Lens V_HZF6_20°	Lens V_HZF6_25°
RV1	[5.81, 3.89, –0.44, 5.00]	[5.43, 4.17, –0.46, 4.97]	[5.03, 4.15, –0.63, 5.42]	[4.42, 3.94, –0.42, 5.82]
RV2	[5.04, 3.98, –0.41, 5.38]	[4.73, 4.20, –0.43, 5.39]	[4.65, 4.22, –0.57, 5.84]	[3.43, 4.01, –0.47, 6.26]
RV3	[3.85, 4.06, –0.51, 6.35]	[4.17, 4.03, –0.42, 6.01]	[3.52, 3.97, –0.50, 6.54]	[2.96, 3.96, –0.48, 6.61]
RV4	[2.44, 3.85, –0.52, 7.33]	[3.59, 3.97, –0.48, 6.45]	[2.59, 3.88, –0.49, 7.07]	[2.20, 3.84, –0.48, 7.47]
RV5	[1.44, 3.66, –0.46, 8.72]	[1.97, 3.77, –0.49, 7.81]	[0.31, 3.51, –0.60, 10.41]	[1.23, 3.49, –0.60, 9.28]

**Table 6:** Predicted lens structural parameters [WD_H_ (mm), CT_H_ (mm), AR_H_ (mm), and PR_H_ (mm)] corresponding to the Ψ and φ values inputted into the INNs of lens H under different RVs.

Cases	Lens H_PC	Lens H_HZF6
RV1	[18.61, 4.55, 8.26, 115.23]	[16.09, 5.41, 10.34, 36.69]
RV2	[18.95, 4.59, 6.54, 129.66]	[18.55, 4.70, 10.07, 202.83]
RV3	[21.96, 4.61, 6.16, 124.76]	[21.21, 5.90, 8.50, 235.52]
RV4	[24.53, 4.53, 5.85, 120.76]	[22.59, 5.95, 7.66, 204.93]
RV5	[26.16, 4.57, 5.54, 117.20]	[25.17, 5.15, 6.45, 136.44]

A statistical metric, known as the mean absolute percentage error (MAPE)^28^, was employed to offer a thorough and quantitative evaluation of the models’ reliability. This metric is defined as $MAPE = \frac{1}{n}\mathop \sum \limits_{1}^{n} \left| {\frac{{y_{i} {-} \hat{y}_{i} }}{{ \hat{y}_{i} }}} \right|$, where *n* represents number of test data, $\hat{y}_{i}$ denotes the lens performance metrics computed by LT, and $y_{i}$ is the performance metrics inputted into the INNs. From the perspective of machine learning, the commonly adopted practice is to maintain a train-to-test data ratio of 8:2. The considerable amount of test data treated in this work may help demonstrate the precision of the proposed INNs for the V and H lenses. The performance metrics of the two lenses in the six test datasets were provided for the models so as to deduce the corresponding lens structural parameters. These parameters were then calculated through LT to derive the simulated performance metrics. The MAPE values of θ for the lens V and Ψ and φ for the lens H were computed to be 0.0265, 0.0263, and 0.1073, respectively. The achieved small MAPE values can categorically prove the substantial congruity between the lens performance metrics obtained from the simulations and the designated performance metrics in the INNs. The low MAPE values confirm the robustness and reliability of the proposed INNs.

## Discussion and outlook

The desired lens performance metrics can be directly inputted into the proposed INNs, which can automatically derive corresponding lens structural parameters and thereby eliminate the need for the conventional trial-and-error process relating to the lens design. Furthermore, the predicted lens parameters have been tested using the commercial design tool, LT. It is discovered that the lens performance metrics obtained by the simulations uphold remarkable correlations between the specified performance values in the INNs. The MSE and MMD associated with the proposed INNs assume practically negligibly low values. Hence, from the perspective of convenience, efficiency, and accuracy of the proposed INNs, the current work is judged to be on a par with the expert-assisted design. The INN-based scheme can be extended to accommodate complex lens systems. In the context of a lens system, each lens component serves a distinct purpose, allowing for individual analysis and design. In this work, we are mainly focused on the design of lenses V and H, aiming to properly adjust the vertical and horizontal fields of view, respectively. The two INNs which are specifically constructed for the lenses V and H can operate independently. Thus, it is feasible to construct multiple INNs for lenses. The independent operating nature of each INN makes it possible to organize the INNs in a cascade fashion, facilitating the inverse design of a system incorporating multiple lenses. The training of the INNs for sophisticated lens systems can be fulfilled through the acquisition of datasets associated with each lens in the lens systems.

In this work, ReLU activation functions are utilized for the proposed INNs. Due to their inherent nonlinearity, ReLU functions can effectively cope with nonlinear mathematical relationships^29–31^, thus facilitating the ability of the proposed INNs to learn and represent intricate functional relationships. In the domain of photonic device design, complex optical characteristics and nonlinear associations manifest frequently. The use of ReLU activation functions enables nonlinear transformation of the input features, thereby enhancing the feature fitting. In specific scenarios where the ReLU function fails to effectively capture the complex nonlinear relationships involved in the optical design, alternative activation functions such as Leaky ReLU^32,33^ and exponential linear units^34,35^ can be relied on to augment the fitting power of the models. Furthermore, the complex and nonlinear relationships can be readily fitted by tuning the hyperparameters of the INNs. It can be asserted that the nonlinear activation functions in conjunction with the hyperparameter tuning help address nonlinear problems, thereby enabling the models to perform precise predictions. Through appropriate training, the INNs hold the potential to expedite the development of a variety of photonic devices involving nonlinear properties, including cases that might have sharp resonant features.

## Conclusion

In this study, we developed a Glow-based INN-driven deep learning technique for the inverse design of optical lenses to satisfy the needs of practical engineering scenarios. The proposed lenses V and H were devised with the objective of narrowing the vertical field of view and expanding the horizontal scanning range. The proposed INNs demonstrated the capability to directly derive lens structures from the specified lens performance metrics. The MSE of the lens performance metrics and MMD of the lens structural parameters obtained through the proposed INNs are approximately 0.001 and 0.025, respectively. The MSE and MMD values in the vicinity of zero serve as evidence of the high accuracy of the proposed INNs. Additionally, the proposed models exhibited high efficiency by delivering lens parameters within 200 ms. Hence, the proposed Glow-assisted INN strategy can simplify and accelerate the inverse design of lenses. Furthermore, our approach is anticipated to enhance the future development of diverse photonic devices, such as waveguide gratings, modulators, and couplers.

## Data Availability

Data is available from the corresponding author upon reasonable request.
